# Characterization, biogenesis model, and current bioinformatics of human extrachromosomal circular DNA

**DOI:** 10.3389/fgene.2024.1385150

**Published:** 2024-04-29

**Authors:** Lina Zhou, Wenyi Tang, Bo Ye, Lingyun Zou

**Affiliations:** ^1^ School of Medicine, Chongqing University, Department of Clinical Data Research, Chongqing Emergency Medical Center, Chongqing University Central Hospital, Chongqing University, Chongqing, China; ^2^ School of Medicine, Jinan University, Guangzhou, Guangdong, China

**Keywords:** extrachromosomal circular DNA, biogenesis model, high-throughput sequencing, bioinformatics pipelines, computational tools

## Abstract

Human extrachromosomal circular DNA, or eccDNA, has been the topic of extensive investigation in the last decade due to its prominent regulatory role in the development of disorders including cancer. With the rapid advancement of experimental, sequencing and computational technology, millions of eccDNA records are now accessible. Unfortunately, the literature and databases only provide snippets of this information, preventing us from fully understanding eccDNAs. Researchers frequently struggle with the process of selecting algorithms and tools to examine eccDNAs of interest. To explain the underlying formation mechanisms of the five basic classes of eccDNAs, we categorized their characteristics and functions and summarized eight biogenesis theories. Most significantly, we created a clear procedure to help in the selection of suitable techniques and tools and thoroughly examined the most recent experimental and bioinformatics methodologies and data resources for identifying, measuring and analyzing eccDNA sequences. In conclusion, we highlighted the current obstacles and prospective paths for eccDNA research, specifically discussing their probable uses in molecular diagnostics and clinical prediction, with an emphasis on the potential contribution of novel computational strategies.

## 1 Introduction

The vast majority of DNA in eukaryotic cells was found on linear chromosomes. In 1965, Yasuo Hotta and Alix Bassel (Hotta and Bassel, 1965) first reported that some DNAs were free from the chromosomal genome and existed in a circular form in wheat embryos and boar spermatozoa. This type of DNA is now referred to as extrachromosomal circular DNA (eccDNA). The revelation that eccDNAs are extensively distributed and can be found in most eukaryotes, including *drosophila* (Stanfield and Lengyel, 1979), yeast (Møller et al., 2015), humans (Cox et al., 1965; Møller et al., 2018), Arabidopsis (Wang et al., 2021), nematodes (Shoura et al., 2017) and pigeons (Møller et al., 2020), has contributed to a better understanding of these molecules.

Early studies on eccDNA were limited by low-throughput experimental techniques, making it difficult for scientists to delve deeper into its biogenesis mechanisms and functions, and thus eccDNA was regarded as a redundant substance far from life processes. Within the past decade or so, with the advancements in high-throughput sequencing and bioinformatics, our understanding of the origin and biological functions of eccDNA has been deepening. A variety of emerging eccDNA detection and analysis tools help people to rapidly discover eccDNAs in tissues and cells, and tap into their connections to other molecules or roles in biological processes, thus redefining their place in the microcosm. For example, significantly frequent amplification of tumor-associated genes has been observed in eccDNAs (Verhaak et al., 2019; Wu et al., 2019; Wang et al., 2021). Furthermore, eccDNAs can act as transcription templates to drive the expression of oncogenes, implying that they play a key role in amplifying cancer signals (Wu et al., 2019). EccDNAs can also mediate genomic evolution in tumors (Nathanson et al., 2014; Luebeck et al., 2023), as well as enhance tumor proliferation and migration (Zou et al., 2024). The findings established eccDNA as a potential biomarker for tumor diagnosis and prognostic assessment. Furthermore, results such as eccDNA containing oncogenes (Kalavska et al., 2018; Gu et al., 2020; Li et al., 2022; Li et al., 2022) and being associated with drug resistance (Alt et al., 1978; Kaufman et al., 1979; Haber and Schimke, 1981; Kaufman et al., 1981; Shoshani et al., 2021) have highlighted the possible use of eccDNA in tumor therapy and monitoring. Freshly, several studies have suggested the use of eccDNA for the diagnosis, treatment, and monitoring of gliomas (Li et al., 2023), gynecologic tumors (Wu et al., 2024), and genitourinary disorders (Lv et al., 2022).

A number of studies provide a thorough summary of current understanding of the functions and roles of eccDNAs, allowing researchers to gain a comprehensive picture of the achievements in experimental science in this area. Wu et al. discussed how eccDNAs lead to drug resistance and accelerate cancer evolution (Wu et al., 2022). Yang et al. meticulously summarized the regulatory mechanisms, and physiological functions of eccDNAs, as well as their roles and potential applications in cancers (Yang et al., 2022). Complementarily, the role of eccDNAs in gynecologic tumors and reproduction has been addressed from a specialized perspective, with the proposal of using eccDNAs as drug targets and biomarkers for non-invasive prenatal testing as well as the early detection, prognosis, and treatment of gynecological tumors (Wu et al., 2024). However, despite the objective contribution of computational methods and tools in advancing eccDNA research, we discovered that there are very few reports that provide a comprehensive summary of this aspect, limiting researchers with a strong interest in eccDNA from understanding the strengths and weaknesses of these tools and making rational choices in their studies.

In this review, we provided a brief summary of the characterization and classification of eccDNAs, their discovery history and biogenesis, with a focus on the tools, computational approaches, and data resources currently available for exploring eccDNAs. We carefully summarized the design, functions, and limitations of various bioinformatics tools, while also discussing the effectiveness and value of these tools in addressing eccDNA-driven tumorigenesis and progression, as well as deciphering other biological functions of eccDNAs, and looking ahead to the potential future directions of bioinformatics for more in-depth eccDNA research.

## 2 Characteristics and diverse capabilities of eccDNA

EccDNAs originate from but are independent of chromosomal DNA, ranging in size from a few hundred to several million base pairs (bp) (Wu et al., 2022). The first record of the presence of extrachromosomal DNA in tumor cases was documented in 1965 (Cox et al., 1965). Subsequently, eccDNAs were isolated from many different organisms and cell types and was given different names: double fragments of chromosome, double minutes (DMs), double bodies, minute chromatin bodies, and accessory chromatin (Wu et al., 2022). In 1990, eccDNA was proposed as an extra-nuclear circular DNA covering all endogenous chromosome sources (Gaubatz, 1990). For a long time, there has been confusion and ambiguity about the naming and classifications of eccDNAs, with variations in the expressions reported in different literature. To avoid confusion caused by inconsistent nomenclature and taxonomy, we synthesized the current understanding of eccDNA and classified them into five types, and described their characteristics and functions individually (Table 1).

**TABLE 1:** The characteristics and functions of different types of eccDNAs.

Categories	Size	Characteristics	Functions
SpcDNA	100bp-10kp	• Mainly contain repetitive genome sequences	• Induce genomic instability
		• Enriched in genetically unstable cells and tissues	• Serve as a marker and an enhancer of genomic instability
MicroDNA	100 bp-400 bp	• Derived from unique non-repetitive genomic regions with high gene density	• Express regulatory RNAs and affect gene expression
		• Enriched in 5′-UTR, exons and CpG islands	• Serve as a non-invasive biomarker to monitor tumor progression and therapeutic efficacy
		• Act as stable, mobile circular DNAs	• May be involved in CNS regulation
Telomeric circles	Integral multiples of 738 bp	• Double-stranded or single-stranded molecules composed of telomeric repeats	• Restore telomere length and maintain the integrity and stability of chromosomes
			• Contribute to cell proliferation
EcDNA	1Mb–3 Mb	• Lack of centromeres and telomeres	• Promote oncogene amplification, tumor evolution and migration
		• Usually carry intact oncogenes	• Contribute to tumor heterogeneity and drug resistance
ERC	19kb–40 kb	• Derived from ribosomal DNA	• Contribute to ribosomal RNA transcription and cellular senescence
		• Able to self-replicate	

### 2.1 Small polydisperse circular DNA (spcDNA)

Small polydisperse circular DNA was initially identified as spcDNA in 1972 after Smith et al. used electron microscopy to detect its presence in HeLa cells (Smith and Vinograd, 1972). At present, eccDNAs that range in size from 100 bp to 10 kp and in a length from 0.2–2.0 μm are referred to as spcDNA. Since repetitive sequences are widely found in spcDNAs (Krolewski et al., 1982; Krolewski et al., 1984; Kunisada and Yamagishi, 1987), it is hypothesized that the majority of spcDNA’s genetic material came from repetitive sequences in the genome (Cohen et al., 1997). Large numbers of direct tandem repeat sequences are favorable for early embryonic cells to produce spcDNAs (Cohen et al., 1997; Cohen and Mechali, 2001). Normal eukaryotic cells have spcDNAs, but genetically unstable cells and tissues, like Hela cell lines, fibroblasts from Fanconi anemia patients, and tumor tissues of colon carcinomas, have higher concentrations of spcDNAs (Cohen and Lavi, 1996; Cohen et al., 1997). Changes in the concentration of spcDNAs may be able to predict genomic destabilization events because increased levels of these molecules have been linked to endogenous and induced genomic instability in human cells (Regev et al., 1998; Wang et al., 2021).

### 2.2 MicroDNA

A novel eccDNA known as microDNA was discovered in mouse and human cell lines by Shibata et al. in 2012 (Shibata et al., 2012). Its size ranges from 100 to 400 bp in size and it originates from unique non-repetitive regions with high gene density. It cannot hold whole gene sequences encoding proteins, but it is rich in the 5′-UTR, exons, and CpG islands. Normal cells in nearly all species, from yeast to humans, have large amounts of microDNAs (Paulsen et al., 2019; Yang et al., 2022). Replication slippage, a replication mistake associated with RNA metabolism or DNA fractures is thought to be its cause (Dillon et al., 2015; Mehanna et al., 2017). It is still unknown, nevertheless, what role microDNAs play in eukaryotic cells. According to a number of recent studies, microDNAs can express functional small regulatory RNAs, including micro RNAs (miRNAs) and novel si-like RNAs, to influence gene expression (Paulsen et al., 2021; Yang et al., 2022). Furthermore, the discovery that tumor cells can release microDNAs into the bloodstream, and the differences and dynamic changes of microDNAs in tumor tissues *versus* normal tissues, as well as in the same tissues of patients before and after surgery (Zhao et al., 2022; Jiang et al., 2023), suggest that microDNAs have the potential to be used as a non-invasive biomarker to monitor tumor progression and therapeutic efficacy (Kumar et al., 2017; Zhu et al., 2018). Smalheiser suggested that microDNA, which is mobile and stable circular DNA, might have a role in regulating memory and communication in CNS neurons (Smalheiser, 2023).

### 2.3 Telomeric circles (t-circles/c-circles)

A unique type of eccDNAs known as telomeric circles are made up only of telomeric repeats and can be either double-stranded (t-circles) or single-stranded molecules (c-circles) (Paulsen et al., 2018). Telomeric circles are thought to have telomerase-like functions that can compensate or restore the shortening of telomere length in chromosomes caused by DNA replication (McEachern et al., 2000; Tomaska et al., 2009; Basenko et al., 2010). Through the telomere lengthening mechanism (ALT) (Reddel, 2003; Mazzucco et al., 2020), they have been revealed to play crucial roles in the immortalization of telomerase-negative malignancies.

### 2.4 ecDNA

DMs have been identified in a range of cancers (Balaban-Malenbaum and Gilbert, 1977; Benner et al., 1991) since they were first noticed in metaphase neuroblastoma cells in 1965. They are created by recombination and head-to-tail concatenation of detachable segments from chromosomes (Cox et al., 1965). DMs, which segregate randomly or asymmetrically during cell division (Lin et al., 1990; Yang et al., 2022), are eccDNAs without telomeres and centromeres that typically carry oncogenes. Kohl et al., for instance, discovered a novel oncogene named MYCN on the DMs of the human neuroblastoma cell line IMR-32 in the 1980s (Kohl et al., 1983). Turner et al. (2017) used a combination of whole-genome sequencing (WGS), structural modeling, and cytogenetic analyses to examine 17 distinct cancer types. Their findings revealed that only 30% of ecDNAs present in tumor cells had characteristics similar to DMs (Turner et al., 2017). As a result, they recommended that the definition of these extrachromosomal particles be expanded. Nowadays, extrachromosomal circular DNAs ranging in size from 1 Mb to 3 Mb are referred to as “ecDNA”, which typically contain complete oncogenes, and are devoid of centromeres and telomeres, and include DMs and their monomeric versions. EcDNAs are essentially nonexistent in normal cells but widely distributed in tumor cells, in contrast to kilobase-size eccDNAs. EcDNA typically exhibits significantly greater oncogene expression than chromosomal DNA (Wu et al., 2019). EcDNA amplification has been shown to be more effective than chromosomal amplification in boosting oncogene copy number and intra-tumor heterogeneity through quantitative analysis of cancer samples (Turner et al., 2017). Moreover, it has been found that ecDNAs have a role in drug resistance (Fan et al., 2011; Yang et al., 2022). Gregory et al. discovered DHFR overexpression on ecDNAs in cancer cells from patients with small-cell carcinoma (SCLC) who received methotrexate (MTX). They found that lowering the amount of ecDNAs in SCLC cells improved MTX sensitivity (Curt et al., 1983; Wang M. et al., 2021). Colon cancer researchers discovered that DHFR-carrying ecDNAs contributed to MTX resistance (Morales et al., 2009; Meng et al., 2015). Lin et al. found that RAB3B amplification of eccDNAs was associated with cisplatin (DDP) resistance in hypopharyngeal squamous cell carcinoma (Lin et al., 2022).

### 2.5 Extrachromosomal rDNA circle (ERC)

ERCs are roughly 19–40 kb in length and can act as templates for ribosomal RNA transcription (Sinclair and Guarente, 1997; Hull and Houseley, 2020). They are formed from ribosomal DNA (rDNA), which is produced through intramolecular homologous recombination of chromosomal DNA. These self-replicating loops, originally found in baker’s yeast, are thought to contribute to its senescence (Sinclair and Guarente, 1997; Nyström, 2007). Mutations in the Sgs1 gene in yeast mother cells have been verified to accelerate senescence (Sinclair et al., 1997). ERCs can accumulate in senescent cells, and Sgs1 mutations promote this accumulation. In contrast, eliminating Fob1 delays the process of ERC accumulation and extends the longevity of yeast mother cells (Defossez et al., 1999).

## 3 Biogenesis model for eccDNA

**FIGURE 1:**
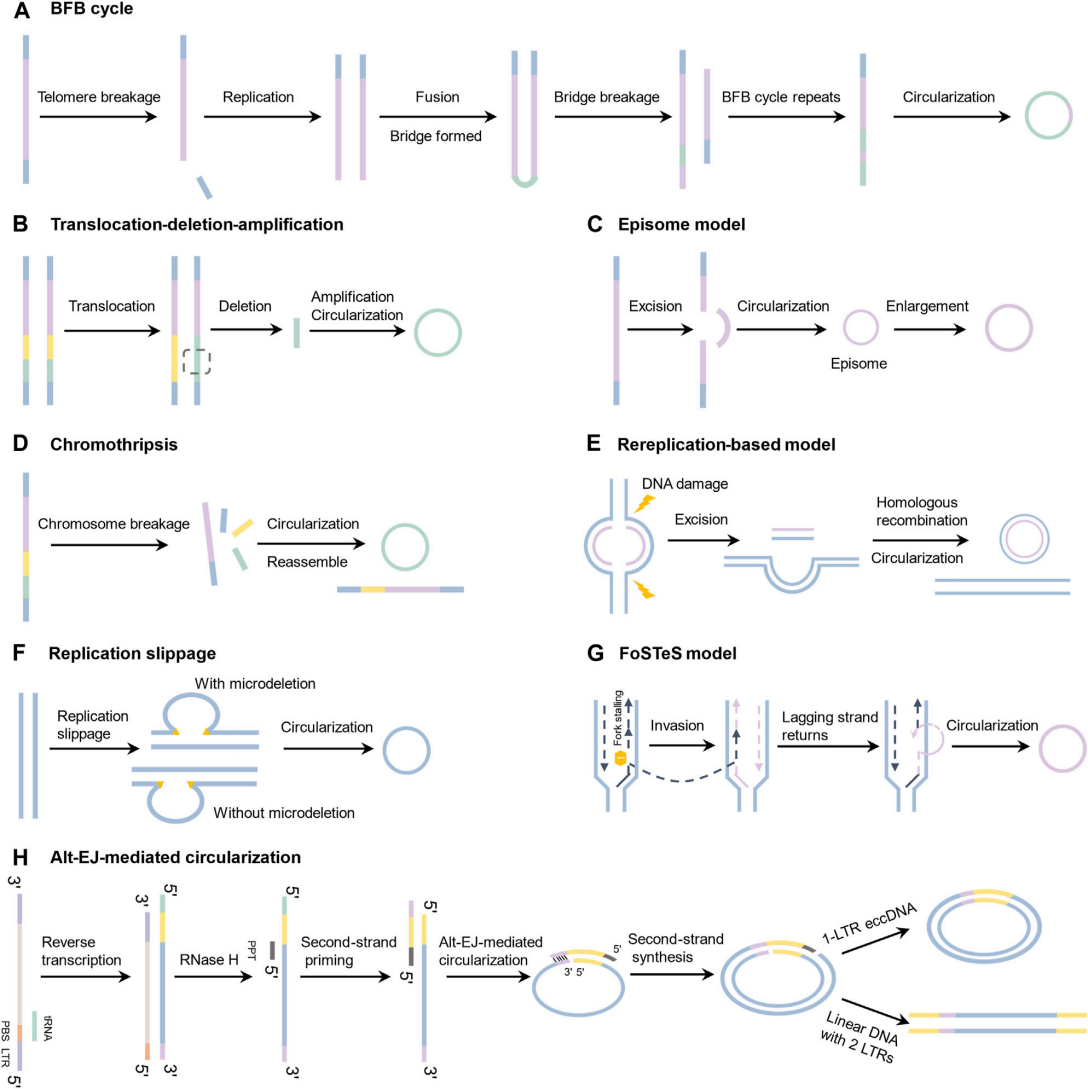
Biogenesis models for eccDNAs based on experimental evidence and extrapolation. **(A)** Breakage-fusion-bridge (BFB); **(B)** Translocation-deletion-amplification; **(C)** Episome model; **(D)** Chromothripsis; **(E)** Rereplication-based model; **(F)** Replication slippage; **(G)** Fork stalling and template switching (FoSTeS) model; **(H)** Alt-EJ-mediated circularization.

Despite their complexity and diversity, eccDNA creation pathways all seem to entail DNA damage. Several alternative processes have been hypothesized, while the precise mechanisms behind the creation of eccDNA are still unknown. Here, we briefly outline various theoretical frameworks elucidating the spontaneous generation of eccDNAs (Figure 1A–G), including breakage-fusion-bridge (BFB) (McClintock, 1938; McClintock, 1941), translocation-deletion-amplification (Van Roy et al., 2006), episome model (Carroll et al., 1987; Carroll et al., 1988), chromothripsis (Stephens et al., 2011; Korbel and Campbell, 2013; Leibowitz et al., 2015), rereplication-based model (Vogt et al., 2004), replication slippage (Dillon et al., 2015), and fork stalling and template switching (FoSTeS) (Lee et al., 2007). According to these broad concepts, eccDNA is associated with apoptosis and is generated through the random bonding of genomic DNA fragments (Wang et al., 2021). The fundamental foundation of these mechanisms is the classic double-strand DNA break repair pathways, which include homologous recombination (HR), non-homologous end joining (NHEJ), and mismatch repair. Few researchers are aware of the repair pathway known as alternative end-joining (alt-EJ). Furthermore, alt-EJ, not HR or NHEJ, was linked to retrotransposon-derived eccDNA biosynthesis in a recent *Drosophila* study (Yang et al., 2023). In order for retrotransposon DNA to generate eccDNA and achieve fresh insertion, alt-EJ stimulates the second-strand synthesis of long terminal repeat (LTR) retrotransposon DNA through a cyclization process (Figure 1H).

### 3.1 Breakage-fusion-bridge (BFB) model

The BFB cycle is one of the most classical eccDNA formation models, proposed by McClintock (Figure 1A) (McClintock, 1938; McClintock, 1941). The BFB cycle begins when a chromosome loses its terminal telomere. If the telomere deletion is not repaired before the chromosome replicates, the chromosome undergoes replication to form two sister chromatids with a single telomere loss. The broken ends of the two sister chromatids then fuse together to produce a dicentric chromosome, which contributes to the creation of an anaphase bridge (Vukovic et al., 2007). The bridge breaks randomly when the dicentric chromosome is dragged to opposite poles. This process proceeds repeatedly, and the telomere-free bridge gradually detaches and circularizes into eccDNAs.

### 3.2 Translocation-deletion-amplification model

The translocation-deletion-amplification scenario involves gene rearrangement around the translocation site (Figure 1B) (Van Roy et al., 2006). When a translocation occurs, DNA segments near the translocation breakpoint may be excised from the chromosome, amplified, and circularized to produce eccDNAs. This model has been validated in a number of experiments, including the co-amplification of the proto-oncogene MYC and the AT motif binding factor 1 (ATBF1) (Van Roy et al., 2006), as well as the co-amplification and overexpression of HMGIC and MDM2 in a carcinoma ex pleomorphic adenoma (Röijer et al., 2002).

### 3.3 Episome model

The episome model is another well-known model of eccDNA production (Figure 1C). EccDNAs are formed by excising DNA segments from chromosomes and amplifying them by recombination. In 1987, Carroll et al. (Carroll et al., 1987) reported the presence of sub-microscopic covalent closed circular DNA outside chromosomes. They established by gel electrophoresis that these molecules are approximately 250–300 kilobase pairs in size, large enough to carry a whole gene, and have been dubbed episomes (Carroll et al., 1987). As a result, scientists investigated the formation mechanism of episomes and developed the episome hypothesis, which proposes that episomes are generated by excising linear DNA from chromosomes, followed by cyclization and amplification (Von Hoff, 1991; Hung et al., 2022). Interestingly, episomes can polymerize into DMs under certain conditions (Carroll et al., 1988; Storlazzi et al., 2006). For instance, Storlazzi and Zuberi discovered that MYC-containing DMs were generated by this model in leukemia cases (Storlazzi et al., 2010; Zuberi et al., 2010).

### 3.4 Chromothripsis model

In this concept, chromosomes break and generate a large number of sequence fragments during a catastrophic event (Figure 1D) (Stephens et al., 2011). Some segments can be ligated randomly by DNA repair mechanisms, resulting in DNA rearrangement clusters. In some situations, DNA fragments can be incorporated into eccDNAs (Zhang et al., 2015; Wu et al., 2022). The hypothesis was proposed in 2011 when Stephens et al. identified dozens to hundreds of genomic rearrangements during a cellular crisis that they dubbed “chromothripsis” (Stephens et al., 2011). A substantial number of complicated chromosomal rearrangements were discovered in Sonic-Hedgehog medulloblastoma (SHH-MB) brain tumors in a patient with Li-Fraumeni syndrome (Rausch et al., 2012). Recent analysis of human cancer WGS data suggested that chromothripsis could be a key factor for speeding genomic DNA rearrangement and amplification into eccDNAs (Cortés-Ciriano et al., 2020; Shoshani et al., 2021).

### 3.5 Rereplication-based model

Vogt et al. used quantitative PCR and chromosomal walking to analyze ecDNAs in gliomas and came up with the Rereplication-based model (Figure 1E) (Vogt et al., 2004). Two breaks occur near the replication eye origins on the same DNA strand, resulting in the excision of double-stranded DNA fragments and the production of eccDNAs. The wound on the chromosome then would be repaired through re-replication (Vogt et al., 2004; Wu et al., 2022).

### 3.6 Replication slippage

In 2015, Dillon et al. isolated and sequenced microDNAs across multiple species and cell types and discovered that the loss of MSH3 DNA mismatch repair protein leads to a significant reduction in microDNA abundance, implying that microDNAs may be generated by mismatch repair following replication slippage (Figure 1F) (Dillon et al., 2015). Specifically, during DNA replication process, polymerase slips on the continuous short direct repetitions, causing DNA loops to form on the product or template strand. The Mismatch Repair (MMR) pathway deletes these DNA circles (Schofield and Hsieh, 2003), but the linked excision products may result in ss microDNAs. Ss microDNA can be converted into ds microDNA by DNA polymerase. Loops on the excised template strand can cause genome microdeletions, but loops on the daughter strand do not.

### 3.7 Fork stalling and template switching (FoSTeS)

The FoSTeS model was originally developed to explain complicated duplication and deletion rearrangements in the dosage-sensitive proteolipid protein 1 (PLP1) gene in patients with Pelizaeus-Merzbacher disease (PMD) (Figure 1G) (Lee et al., 2007). Afterward, Yang et al. examined ecDNA breakpoints and proposed that fork stalling and template switching during DNA replication could be another source of ecDNAs (Yang et al., 2013). In this model, replication forks stall at the damage site, and the lagging strand separates from the current template and invades a nearby template strand to synthesize new DNA. FoSTeS events can occur multiple times in a single replication. Due to the poor complementarity between the synthesized daughter strand and the starting template during template switching, DNA bulges may develop on either strand (Lee et al., 2007; Wu et al., 2022), potentially resulting in the formation of eccDNA (Dillon et al., 2015).

### 3.8 Alt-EJ-mediated circularization

Yang and colleagues discovered that only 10% of retrotransposons were able to undergo insertion, with the remaining 90% existing as eccDNAs (Yang et al., 2023). Through a genetic screening approach, they identified alt-EJ as a less common DNA repair mechanism that plays a role in retrotransposon insertion and the generation of retrotransposon-derived eccDNA structures (Figure 1H). In this proposed model, the RNA molecule is initially reverse transcribed to generate the first-strand DNA, followed by digestion of the RNA component in the DNA-RNA hybrid by RNase H, leaving the polypurine tract (PPT) intact at the 3′end. The PPT acts as a primer for the synthesis of the long terminal repeat (LTR) and primer binding site (PBS) sequences necessary for the formation of the second DNA strand. Subsequently, alt-EJ facilitates the circularization of the two DNA strands by aligning their PBS homology regions. This circularization event triggers the initiation of second-strand DNA synthesis, resulting in the formation of a non-covalent loop structure. Depending on the subsequent processing, this loop can either be filled to generate a covalent 1-LTR eccDNA or lead to the formation of linear DNA containing two LTRs.

## 4 Computational identification and prediction for eccDNA

**FIGURE 2:**
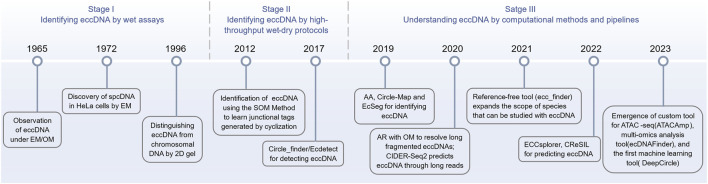
Timeline of the evolution of the eccDNA research tools (from traditional experimental techniques to advanced computational approaches).

Although eccDNA has been studied throughout the 20th century, technological limitations at the time prevented researchers from digging it. Only in recent years, the advances in isolation, purification, and sequencing, as well as the lightning-fast development of computer arithmetic, storage, and algorithms, allowed researchers to investigate the mysteries of eccDNA in a broader range of species and materials. The computational detection and analysis tools dedicated to eccDNA have revealed a diverse and blossoming landscape (Figure 2), which is worth summarizing in a timely manner.

### 4.1 Imaging-based visualization methods

Prior to the development of sequencing technologies, microscopic imaging was instrumental in the discovery and characterization of eccDNAs. EccDNAs were first discovered using electron microscopy (EM) (Hotta and Bassel, 1965), although optical microscopy (OM) can also be used to detect circular DNA (Cox et al., 1965). However, due to resolution limitations, OM can only detect big molecular weight eccDNAs (DMs). In 2019, Wu et al. validated the circular structure of eccDNAs by using super-resolution three-dimensional structured illumination microscopy (3D-SIM) (Wu et al., 2019). In other studies, scanning electron microscopy (SEM) and transmission electron microscopy (TEM) were used with molecular localization techniques such as fluorescence *in situ* hybridization (FISH) to track individual eccDNAs (Koo et al., 2018). Furthermore, Yi et al. (Yi et al., 2022) used the CRISPR-based ecTag approach to fluorescently label eccDNAs in living cells and monitor their heterogeneous segregation during mitosis. Two-dimensional (2D) gels are also useful for distinguishing the structural properties of eccDNAs (Cohen and Lavi, 1996; Peng et al., 2022).

The application of computer vision algorithms has significantly enhanced eccDNA visualization as well as detection and analysis efficiency. Ecdetect (Starling, 2017) and ecSeg (Rajkumar et al., 2019) are powerful image analysis programs that identify and quantify eccDNAs. ECdetect is a kind of semi-automated image analysis software that can be used alongside whole genome sequencing (Starling, 2017). Turner et al. used ECdetect to detect and analyze ecDNAs in 2,572 cells from various cancer types, and found that ecDNAs exist in nearly half of human cancers (Turner et al., 2017). Nevertheless, ECdetect may overlook eccDNAs due to its low sensitivity. EcSeg is a U-network-based tool that precisely measures ecDNAs from images stained with 4′,6-diamidino-2-phenylindole (DAPI) and integrates FISH signals to pinpoint amplified oncogenes on ecDNA (Rajkumar et al., 2019).

However, imaging-based approaches have the following limitations: i) Live cells must be cultured in order to observe stained cells at metaphase; ii) Some eccDNAs are too small to be observed even after staining; iii) Only fluorescent probe-labeled eccDNAs can be detected after hybridization with a specific probe, which still does not allow for sequence characterization; and iv) Low throughput.

### 4.2 High-throughput sequencing-based assays

High-throughput sequencing technology, along with bioinformatics pipelines, has created a new strategy for detecting, structurally validating, and functionally identifying eccDNAs, resulting in the emergence of a plethora of tools (Table 2). In general, short-read-based tools were created to identify high-confidence eccDNAs using data such as breakpoints, split reads, discordant read pairs, read depths, and soft-clip realignment. They are extensively used because of their low cost, high accuracy, and support for short reads from Illumina platforms, including Circle_finder (Kumar et al., 2017), AmpliconArchitect (Deshpande et al., 2019), Circle-Map (Prada-Luengo et al., 2019) and ECCsplorer (Mann et al., 2022), among others. Because long reads are not supported, this technique fails to recognize major structural rearrangements, makes full-length eccDNAs construction problematic, and increases the possibility of information loss. To address these challenges, long-read tools based on third-generation sequencing (TGS) technologies, such as CIDER-Seq2 (Mehta et al., 2020), ecc_finder (Zhang et al., 2021), CReSIL (Wanchai et al., 2022) and Flec (Wang et al., 2023), have emerged to support raw data from the PacBio or Nanopore platforms. They get the full-length consensus sequence of eccDNAs through *de novo* assembly, which aids in the identification and characterization of eccDNAs as well as the discovery of their origins, production process, and immunostimulatory functions (Wang Y. et al., 2021).

**TABLE 2:** High-throughput sequencing-based assays for eccDNA identification.

Name	Reads type	Reference free	Repeated loci considered	EccDNA enrichment needed	Suitable for giant genomes	Mapping aligner	URL	References
AmpliconArchitect	short-read	NO	NO	NO	NO	BWA	https://github.com/virajbdeshpande/AmpliconArchitect	Deshpande et al. (2019) (Deshpande et al., 2019)
AmpliconReconstructor	short-read	NO	NO	NO	YES	BWA	https://github.com/AmpliconSuite/AmpliconReconstructorOM	Luebeck et al. (2020) (Luebeck et al., 2020)
Circle_finder	short-read	NO	NO	NO	NO	Novoalign	https://github.com/pk7zuva/Circle_finder	Kumar et al. (2017) (Kumar et al., 2017)
Circle-Map	short-read	NO	YES	YES	NO	BWA	https://github.com/iprada/Circle-Map	PradaLuengo et al. (2019) (Prada-Luengo et al., 2019)
CIDER-Seq2	long-read	NO	NO	YES	YES	Muscle	https://github.com/devang-mehta/ciderseq2	Mehta et al. (2020) (Mehta et al., 2020)
ECCsplorer	short-read	YES	NO	YES	YES	segemehl	https://github.com/crimBubble/ECCsplorer	Mann et al. (2022) (Mann et al., 2022)
ecc_finder	short-read and long-read	YES	YES	YES	YES	minimap2&BWA	https://github.com/njaupan/ecc_finder	Zhang et al. (2021) (Zhang et al., 2021)
CReSIL	long-read	NO	YES	YES	YES	—	https://github.com/visanuwan/cresil	Wanchai et al. (2022) (Wanchai et al., 2022)
ATACAmp	short-read	NO	YES	NO	YES	BWA	https://github.com/chsmiss/ATAC-amp	Chang et al. (2023) (Cheng et al., 2023)

In WGS data, the copy number of eccDNAs increases with cell proliferation, and high-throughput-based technologies tend to overlook many low-frequency eccDNAs and are susceptible to interference from chromosomal DNA. Therefore, targeted processing of sequences prior to sequencing is crucial. Circle-seq (Møller et al., 2015) is a sensitive, genome-wide approach for enriching, purifying, and detecting eccDNAs. First, it employs column chromatography to separate denatured eccDNA from linear DNA in damaged cells. The remaining linear DNA is digested by exonuclease. Later, the circular DNA is enriched by φ29 rolling circle amplification (RCA). Finally, the eccDNAs were identified by high-throughput sequencing and eccDNA detection tools. CIDER-Seq (Mehta et al., 2020) is another novel method for sequencing circular DNA. It uses randomly primed circular DNA amplification, followed by single-molecule sequencing to get high-quality reads. The accuracy of CIDER-Seq-generated eccDNA varies with sequence length, with the best accuracy when the molecules are smaller than 10 kb (Mehta et al., 2020). An alternate approach is to identify eccDNA from tumor genomes using ATAC-seq (Kumar et al., 2020; Su et al., 2021; Cheng et al., 2023). Kumar et al. found more than 18,000 eccDNAs when they performed a pan-cancer analysis of ATAC-seq libraries from 23 tumor types (Kumar et al., 2020). Cheng et al. created an algorithm called “ATACAmp”, which uses anomalous reads from ATAC-seq to identify eccDNA breakpoint locations and amplified regions (Cheng et al., 2023). Circle_finder, a previously disclosed program, suggests highly likely eccDNAs by analyzing ATAC-seq datasets (Kumar et al., 2017; Su et al., 2021).

**FIGURE 3:**
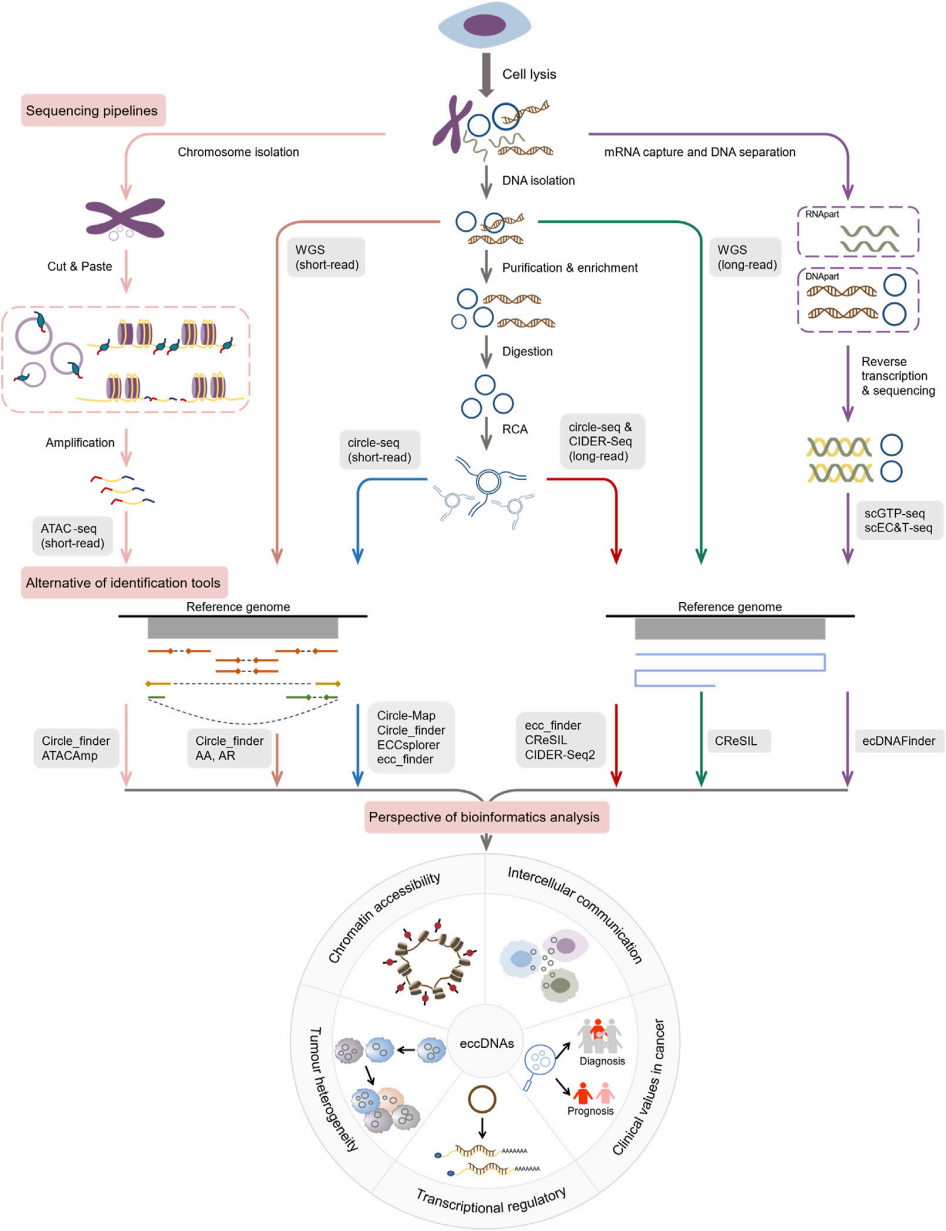
Current pipelines and future hotspots for exploring eccDNAs by bioinformatics.

It is a challenge to select the most appropriate toolsets from among many accessible to further the research. After identifying a tool, it is critical to consider its possible applications and further research avenues. To improve the efficiency of researching eccDNA, we conducted a systematic overview (Figure 3) here. i) AmpliconArchitect (AA) and AmpliconReconstructor (AR). AA is a powerful toolkit for extracting eccDNA signals from short-read WGS data. AA algorithm begins with seed intervals and mapped reads and implements a series of steps containing amplicon interval search, CNV boundary detection, breakpoint graph construction, cyclic decomposition of the graph, and interactive merging of the cycles, to reconstruct the possible structure of eccDNAs and other amplicons (Deshpande et al., 2019). Chapman et al. employed the AA algorithm to detect the presence of eccDNAs in 18% cases from WGS data of 468 medulloblastomas (Chapman et al., 2023), demonstrating its reliability. In Deshpande’s benchmarking, AA had an error rate of <11% (Deshpande et al., 2019). However, for complex amplicons like BFBs, AA may fail to capture their fine structures, leaving various possible reconstruction options (Jiang et al., 2023). As a result, Luebeck’s team developed the AmpliconReconstructor (AR) (Luebeck et al., 2020), based on AmpliconArchitect, which enables OM of large fragments (>150 kb) using NGS to resolve eccDNA at single-nucleotide resolution. It can disambiguate numerous junctions for more specific and accurate amplicon reconstruction. Researchers used AR to reconstruct fCNAs in seven cancer cell lines, revealing the complicated structures of ecDNAs, BFBs and others (Luebeck et al., 2020). ii) Circle_finder. Circle_finder is intended for analyzing Illumina short reads. It can analyze WGS data and also can predict eccDNAs from circle-seq and ATAC-seq data. Kumar et al. exploited the tool and discovered that microDNAs in serum and plasma samples from the same individuals would be shorter than in samples collected before surgery weeks following tumor resection, which is consistent with the fact (Kumar et al., 2017). It is hypothesized that eccDNAs in circulation may complement miRNA and linear DNA, potentially serving as a means for disease diagnosis and intercellular communication (Kumar et al., 2017). Unfortunately, Circle_finder cannot discriminate between eccDNA and chromosomal segmental tandem repeats (Zhao et al., 2023; Wu et al., 2024). iii) Circle-Map. Circle-seq (Møller et al., 2015), together with Circle-Map (Prada-Luengo et al., 2019), is widely used for eccDNA characterization. The method utilizes a Position Specific Scoring Matrix (PSSM) to develop an alignment probability model for aligning reads at eccDNA breakpoint junctions. Circle-Map is more sensitive than Circle_finder in detecting circular DNA in both simulated and real datasets with a high degree of precision (Prada-Luengo et al., 2019). Circle-Map exceeded Circle-finder by 17% in the simulated dataset with 30× coverage, with a sensitivity of 0.943 and a precision > 0.97. iv) CIDER-Seq2. CIDER-Seq2 is a customized data analysis package to parse CIDER-Seq using the DeConcat algorithm. CIDER-Seq employs PacBio long-read sequencing to generate full-length sequences, which are then de-emasculated by DeConcat. The filtered DeConcat output is then sent into the eccDNA detection module to select *bona fide* circular sequences. This program aligns these sequences to the reference genome in order to determine the genomic origin of each detected eccDNA. However, this method requires a high level of computer performance (Mehta et al., 2020). v) ECCsplorer. Unlike the bioinformatics pipelines discussed above, which all require reference genomes, ECCsplorer is more compatible and appropriate as a sort of software with or without reference genomes for non-model organisms (Mann et al., 2022). It comprises a mapping module and a clustering module. In the mapping module, firstly, possible circular regions with at least five split reads are identified using the haarz (Hoffmann et al., 2014) algorithm; secondly, regions containing at least one discordant pair are identified via SAMtools (Li et al., 2009) and BEDTools (Quinlan and Hall, 2010); thirdly, high coverage regions of eccDNAs are detected according to SciPy’s peak finder algorithm (Virtanen et al., 2020). The clustering module compares circle sequences with unbenchmarked control data using RepeatExplorer2 (Novák et al., 2010) to discover specifically enriched DNA circles. The two programs can be run independently or jointly (Mann et al., 2022). The pipeline’s main constraints are the implemented tools and algorithms, which developers suggest can be solved by partitioning the dataset and executing it several times (Mann et al., 2022). Perhaps upgrading the mapping aligner could improve the tool’s performance significantly. In a study, researchers evaluated the sensitivity of four tools (Circle-Map, Circle_finder, ECCsplorer and ecc_finder) using eccDNA datasets from the human and wheat genomes, and found that the indexing time of segemehl used by ECCsplorer is double that of BWA (Zhang et al., 2021). Anyway, ECCsplorer may be a viable option for processing non-modeling organisms and low-coverage, short-read sequencing data. vi) Ecc_finder. Ecc_finder is a comprehensive and reference-free tool dedicated to detecting eccDNAs from Illumina short reads and Nanopore long reads. Zhang et al. used eccDNA data from *Homo sapiens*, heat-stressed *Arabidopsis thaliana*, and common wheat to test the sensitivity, accuracy, and computational time of Circle-Map, Circle_finder, ECCsplorer and ecc_finder under the default model. They found that ecc_finder improved in each of these areas (Zhang et al., 2021). Compared to other tools, ecc_finder may be the best choice for really big genomes. vii) CReSIL. CReSIL is a set of tools for identifying and localizing eccDNAs based on RCA (Wanchai et al., 2022). It identifies and characterizes eccDNAs from long reads and also provides function annotation and Circos to visualize eccDNAs. CReSIL also recognizes eccDNAs from long-read WGS, but is unable to capture eccDNAs originating outside the reference genome and has difficulty detecting eccDNAs smaller than 200 nt (Wanchai et al., 2022). Furthermore, CReSIL requires extensive sequence coverage. CReSIL accurately detects eccDNA in whole genome long-read sequencing data, achieving a minimum F1 score of 0.98 at high coverage depths (100×, 50×, and 20×) (Wanchai et al., 2022). Nonetheless, decreased coverage depth will have a negative impact on CReSIL’s performance. viii) ATACAmp. ATACAmp recognizes the open DNA features of eccDNAs in ATAC-seq data, allowing for a more efficient and cost-effective prediction of these genomic variation (Cheng et al., 2023). It advances our understanding of the complicated interplay between chromatin accessibility of eccDNAs and genomic structure in cancer. Currently, ATACAmp does not implement filtering of some blacklist regions, which will be optimized in future iterations to increase accuracy.

### 4.3 Single-cell multi-omics-based pathways

Single-cell multi-omics technologies enable the integration of information from different sequencing modalities, resulting in a more accurate correlation between genotypes and phenotypes (Macaulay et al., 2017). These approaches have revealed the regulation, function, and mechanism of critical molecules in disease pathogenesis, leading to the discovery of new diagnostic markers and therapeutic targets. Several approaches have been reported to be capable of single-cell multi-omics analysis for eccDNA genomes and transcriptomes, including scEC&T-seq (Chamorro González et al., 2023) and scGTP-seq (Chang et al., 2023). The scEC&T-seq approach isolates mRNA and DNA from single cells by magnetic bead way, followed by independent amplification and sequencing using the Smart-seq2 and scCircle-seq workflows. González et al. used ScEC&T-seq in cancer cells to capture differences in eccDNA abundance between cells, resolve structural heterogeneity, and explore implications for transcriptional regulation. They identified eccDNA clones containing oncogenes in cancer cells, which led to differences in intercellular oncogene expression (Chamorro González et al., 2023). The scGTP-seq workflow established by Chang et al. involves sequencing and analysis of separated DNA and RNA via scWGA and Smart-seq2 (Chang et al., 2023). They further developed ecDNAFinder for deciphering eccDNA in scGTP-seq. FISH reverse validation demonstrated that the combination of scGTP-seq and ecDNAFinder was able to identify endogenous eccDNA (Chang et al., 2023). Of course, eccDNAs can also be validated by PCR and Sanger sequencing (Wu et al., 2019; Fan et al., 2021; Yang et al., 2022). Estimating intratumoral heterogeneity based on single-cell multi-omics sequencing data from medulloblastoma cells, copies of eccDNAs show intercellular variability, which may contribute to tumor heterogeneity (Chapman et al., 2023; Zhao and Verhaak, 2024). Therefore, we believe such approaches will shed more light on the novel role of eccDNA elements in the origin and evolution of cancer cells.

### 4.4 Machine learning-based approaches

The utilization of machine learning presents new opportunities for the investigation of eccDNA. The isolation of eccDNA from linear DNA using deep learning models, such as DeepCircle, a bioinformatics framework developed by Chang et al. (Chang et al., 2023), has been shown to improve the accuracy of eccDNA identification. DeepCircle, the pioneering machine learning model for predicting the presence of eccDNA, leverages convolutional neural network (CNN) and Bidirectional Encoder Representations of Transformers (BERT) built on attention mechanisms to forecast eccDNAs. This model has demonstrated strong predictive performance, achieving an accuracy level of approximately 80%. Through its interpretation module, DeepCircle scrutinizes and assimilates sequence characteristics from a substantial number of eccDNA samples to predict consensus eccDNA-related motifs. The discovery of these consensus motifs offers insight into elucidating the formation mechanism and biological function of eccDNAs. Furthermore, novel disease models have emerged that combine eccDNA signatures with clinical data to assess the correlation of eccDNA expression with disease and predict clinical outcomes. For instance, Li et al. (Li et al., 2023) established a three-stage model for gliomas based on eccDNA-carrying genes, employing hundreds of machine learning algorithms and stacked ensemble modeling for clinical diagnosis, prognostic prediction, and recurrence risk prediction. Despite the advancements in machine learning-based tools, there remains a necessity for comprehensive training datasets to refine the model and enhance its adaptability and robustness in diverse scenarios.

## 5 Online resources for exploring eccDNA

In the current era of high-throughput technologies, research on eccDNAs has significantly expanded, resulting in the accumulation of vast amounts of raw data. However, despite the abundance of literature and sequencing data available, knowledge regarding the properties and functions of eccDNAs remains fragmented. To address this issue, efforts have been made to organize the data and establish databases for information integration. This study has compiled and compared various online databases dedicated to eccDNAs, such as CircleBase (Zhao et al., 2022), eccDNAdb (Peng et al., 2022), eccDNA Atlas (Zhong et al., 2023), EccBase (Sun et al., 2023), and eccDB (Yang et al., 2023), in terms of their design and functionality (Table 3). For instance, CircleBase, as the pioneering eccDNA database, provides comprehensive annotations of 601,036 eccDNAs derived from the human genome. By scoring each eccDNA, CircleBase helps to analyze their potential functions in the human genome and predicts regulatory networks between eccDNAs and the epigenetics. On the other hand, eccDNAdb is a specialized tumor eccDNA database that catalogs eccDNA genes in tumor tissues or cell lines, particularly in conditions like glioblastoma, gastric cancer, and ovarian cancer. Unlike CircleBase, eccDNAdb places greater emphasis on investigating the correlation between identified eccDNA genes and tumor expression or prognosis. Meanwhile, eccDNA Atlas stands out as the most extensive repository of eccDNA sources, encompassing information on eccDNAs from seven species, including *Homo sapiens, Arabidopsis thaliana, Drosophila, Mus musculus, Cricetulus griseus, Yeast, and Gallus gallus domesticus*. EccBase boasts the largest collection of eccDNAs to date, with over 1.2 million entries which organized by tissue and cell line types, offering insight into identified or predicted eccDNAs in humans and mice. Additionally, apart from standard functions like searching, browsing, analyzing, and downloading data, the latter two databases also feature a BLAST module, enabling users to discover and annotate novel candidates through similarity alignment and homology identification. Notably, eccDB distinguishes itself by its unique ability to analyze the interactions within and between eccDNA chromosomes.

**TABLE 3:** Comparison of current available eccDNA databases.

Database	Species of origin	Number of eccDNA entries	Predicted eccDNA contained	Function				Website	References
				Annotate	Analyse	blast	Submit		
CircleBase	*Homo sapiens*	601,036	YES	✓	✓	✗	✗	http://circlebase.maolab.org	Zhao et al. (2022b)
eccDNAdb	*Homo sapiens*	1270	YES	✓	✓	✗	✓	http://www.eccdnadb.org	Peng et al. (2022b)
eccDNA Atlas	*Homo sapiens, Arabidopsis thaliana, Drosophila*, *Mus musculus, Cricetulus griseus, Yeast, Gallus gallus domesticus*	639,313	YES	✓	✓	✓	✓	http://lcbb.swjtu.edu.cn/eccDNAatlas	Zhong et al. (2023)
EccBase	*Homo sapiens, Mus musculus*	1235772	YES	✓	✓	✓	✓	http://www.eccbase.net	Sun et al. (2023)
eccDB	*Homo sapiens, Arabidopsis thaliana, Mus musculus, Saccharomyces Cerevisiae*	767,981	YES	✓	✓	✓	✗	http://www.xiejjlab.bio/eccDB	Yang et al. (2023b)

## 6 Discussion and prospects

In this overview, we consolidate the latest advances in eccDNA research, focusing on the contributions of bioinformatics tools and resources to the field. We discuss the significance of utilizing appropriate tools for eccDNA molecule identification following high-throughput sequencing, as well as the establishment of reliable experimental pipelines and computational models to enhance our understanding of the biological and pathological roles of eccDNAs. These current and prospective research directions underscore the importance of leveraging bioinformatics in advancing eccDNA studies, and we provide concise recommendations to guide future investigations in this area.

Numerous unexplored inquiries persist within this field. For instance, there remains a lack of direct experimental evidence to substantiate the hypothesized mechanisms of eccDNA formation and maintenance. Questions arise regarding the fate of eccDNA once it is established: what is its ultimate fate? Can it be degraded through cellular-related mechanisms to maintain genomic stability, or can it reintegrate into chromosomes? And can it be synthesized *in vitro* and serve as plasmid-like vectors carrying useful genes into host cells? Presently, various lines of evidence suggest that eccDNAs have the potential to be targets for cancer treatment, as the removal of tumor eccDNAs has been shown to diminish oncogene amplification (Huh et al., 2016; Deshpande et al., 2019; Yerlici et al., 2019; Arrey et al., 2022). Regrettably, the majority of these studies have been confined to cellular and animal models, leaving the question of how to target eccDNAs in human tumor cells unanswered. Furthermore, while it is known that eccDNAs are linked to genomic instability, their specific topology of eccDNAs remains undisclosed.

Emerging fields heavily rely on the development of innovative tools to facilitate new discoveries. Current research on eccDNAs predominantly utilizes NGS/TGS. However, there is a notable absence of a universally accepted standard or guideline for conducting studies in this area, leading to potential discrepancies in results when employing different methodologies. For instance, the commonly used experimental techniques such as circle-seq and computational software like AA may also lead to information missing, false positives and difficulties in resolving complex amplicon structures (Møller et al., 2015; Deshpande et al., 2019). Many existing tools face challenges in characterizing complex or large eccDNA, and need to reduce the false-positive rate, enhance accuracy, improve time efficiency for large-scale processing, and be able to apply to different experimental scenarios. The emergence of deep learning and large models presents promising avenues to tackle these challenges. Deep learning algorithms possess robust capabilities in dimensionality reduction and feature mining, particularly when coupled with large-sample multi-omics data, enabling sufficient training of predictive models to significantly improve the prediction accuracy of eccDNA and its functional roles. The well-trained prediction algorithms can be applied to identify cancer-related eccDNA consensus sequences, thereby facilitating the discovery of potential eccDNA biomarkers across various cancers. Furthermore, deep learning-based macro models are adept at integrating intelligence from the literature, unstructured experimental data (e.g., images), and databases to gain profound insights into the intricate relationship between eccDNA and the microcosm from diverse perspectives. This holistic approach holds the promise of establishing a comprehensive understanding of disease for eccDNA investigation akin to the realization of WYSIWYG-style pathways.

Several intriguing research avenues for eccDNA warrant further investigation in the future. One such area of interest involves elucidating the physicochemical nature of eccDNA in driving cancer evolution, heterogeneity, and drug resistance to therapeutics. This requires a combination of tumor spatiotemporalomics, single-cell holography, live-cell imaging, and computational modeling to analyze the myriad of data to uncover how eccDNA facilitates tumor proliferation, immune evasion, and treatment resistance. Current bioinformatics tools are clearly unable to meet these needs, highlighting the need for novel algorithms capable of processing multimodal data inputs while maintaining high interpretability. Another promising research direction is untangling the intricate interplay between eccDNAs and genomes, pathways, environments, diseases, and drugs. This involves constructing knowledge graphs or data pivot tools to realize the correlation between various information sources, designing tools to identify specific eccDNAs from disease entity samples, and utilizing micro-enrichment kits and rapid sequencing to develop new hematological diagnostic methods for early disease detection and treatment monitoring. Additionally, there is a need to establish efficient experimental and computational pipelines to explore the vulnerabilities of cells harboring eccDNAs and their potential to trigger immune system responses. Strategies and techniques must be devised to target these eccDNAs, paving the way for innovative eccDNA-targeted immunotherapies. Lastly, enhancing existing tools or creating new ones to screen eccDNAs with therapeutic potential in patients and validate corresponding antibodies or chemical drugs, and establish a comprehensive disease treatment framework centered around eccDNA represents a critical research objective.

There is a considerable distance yet to be covered in the translation of eccDNA research into clinical applications. An example of a current challenge is the unsuitability of differential eccDNA in tissues for testing purposes. Therefore, it is imperative to continue conducting extensive experiments to demonstrate the disease-specific expression of eccDNA in plasma or other bodily fluids or excreta, and subsequently quantify its abundance, half-life, molecular weight, and other relevant characteristics. Validation of these eccDNAs in large-scale human and animal samples is necessary to identify potential biomarkers. Subsequent development of kits to enrich these molecules for rapid analysis using standard laboratory equipment is essential. Furthermore, it is crucial to address population-specific differences in this context. For instance, while patients in the early stages of cancer have been found to release minute amounts of specific eccDNA into plasma, these levels must be consistently detectable at lower thresholds and remain stable across diverse populations. The current body of evidence from randomized controlled trials in this field is insufficient, and the significance of an identified eccDNA marker as a disease indicator necessitates long-term prospective studies for confirmation. Despite the challenges ahead, there is optimism that eccDNA research will transition from laboratory settings to clinical practice.

In summary, advancements in eccDNA identification and prediction are propelled by a combination of experimental innovations and algorithm ingenuity. Due to the complexity and breadth of this field, a comprehensive overview and future outlook are beyond the scope of this discussion, and anticipation is held for further remarkable discoveries in eccDNA bioinformatics.

## Data Availability

The original contributions presented in the study are included in the article/Supplementary material, further inquiries can be directed to the corresponding author.
